# The Impact of Ozone on Periodontal Cell Line Viability and Function

**DOI:** 10.3390/cimb47020072

**Published:** 2025-01-23

**Authors:** Nada Tawfig Hashim, Rasha Babiker, Shahistha Parveen Dasnadi, Md Sofiqul Islam, Nallan CSK Chaitanya, Riham Mohammed, Nancy Soliman Farghal, Bakri Gobara, Muhammed Mustahsen Rahman

**Affiliations:** 1 Department of Periodontics, RAK College of Dental Sciences, UAE Sciences, RAK Medical & Health Sciences University, Ras Al Khaimah 12973, United Arab Emirates; mustahsen@rakmhsu.ac.ae; 2 Department of Physiology, RAK College of Medical Sciences, RAK Medical & Health Sciences University, Ras Al Khaimah 11172, United Arab Emirates; rashababiker@rakmhsu.ac.ae; 3 Department of Orthodontics, RAK College of Dental Sciences, UAE Sciences, RAK Medical & Health Sciences University, Ras Al Khaimah 12973, United Arab Emirates; shahistha.parveen@rakmhsu.ac.ae; 4 Department Operative Dentistry, RAK College of Dental Sciences, UAE Sciences, RAK Medical & Health Sciences University, Ras Al Khaimah 12973, United Arab Emirates; sofiqul.islam@rakmhsu.ac.ae; 5 Department of Oral Medicine & Radiology, RAK College of Dental Sciences, UAE Sciences, RAK Medical & Health Sciences University, Ras Al Khaimah 12973, United Arab Emirates; krishna.chytanya@rakmhsu.ac.ae; 6 Department of Oral Surgery, RAK College of Dental Sciences, UAE Sciences, RAK Medical & Health Sciences University, Ras Al Khaimah 12973, United Arab Emirates; riham.abdelraouf@rakmhsu.ac.ae; 7 Department of Endodontics, RAK College of Dental Sciences, UAE Sciences, RAK Medical & Health Sciences University, Ras Al Khaimah 12973, United Arab Emirates; nancy.soliman@rakmhsu.ac.ae; 8 Department of Oral Rehabilitation, Faculty of Dentistry, University of Khartoum, Khartoum 11115, Sudan; bakrigobara10@gmail.com

**Keywords:** O_3_ therapy, periodontal disease, antimicrobial, inflammation modulation, tissue regeneration, periodontal cell lines

## Abstract

Periodontal diseases, including gingivitis and periodontitis, are chronic inflammatory conditions of the teeth’ supporting structures that can lead to progressive tissue destruction and loss if left untreated. Basic treatments like scaling and root planing, alone or combined with antimicrobial agents, are the standard of care. However, with the increasing prevalence of antibiotic resistance and the need for new ideas in therapy, adjunctive treatments like ozone therapy have gained attention. Ozone (O_3_), a triatomic oxygen molecule, is used because of its strong antimicrobial, anti-inflammatory, and regenerative activity and, hence, as a potential tool in periodontal therapy. This review of the use of ozone therapy in periodontal disease breaks down the multifaceted mechanism of ozone therapy, which includes the selective antimicrobial action against biofilm-associated pathogens, immunomodulatory effects on host cells, and stimulation of tissue repair. O_3_ therapy disrupts microbial biofilms, enhances immune cell function, and promotes healing by activating Nuclear Factor Erythroid 2-Related Factor 2 (Nrf2) and Mitogen-Activated Protein Kinase (MAPK) signaling pathways that regulate oxidative stress, inflammation, and apoptosis. Additional findings include its ability to upregulate growth factors and extracellular matrix proteins, which is significant for periodontal tissue regeneration. This review also discusses the application of O_3_ therapy in periodontal cell lines, emphasizing its impact on cell viability, proliferation, and differentiation. Advances in periodontal regenerative techniques, combined with the antimicrobial and healing properties of O_3_, have demonstrated significant clinical benefits. Challenges, including the need for standardized dosages, effective delivery systems, and long-term studies, are also addressed to ensure safe and effective clinical integration. O_3_ therapy, with its dual antimicrobial and regenerative capabilities, offers an innovative adjunctive approach to periodontal treatment. Future research focusing on optimized protocols and evidence-based guidelines is essential to fully realize its potential in enhancing periodontal health and improving patient outcomes.

## 1. Introduction

Periodontal diseases, including gingivitis and periodontitis, are a class of oral health issues that impact the supporting structures of the teeth, including the periodontal ligament, cementum, and alveolar bone [1]. Periodontitis is a chronic inflammatory condition, usually of bacterial etiology, resulting in the gradual loss of the soft tissues and bone that support the teeth. Periodontitis may result in permanent tissue deterioration if ignored or insufficiently treated, potentially causing tooth mobility and, eventually, tooth loss. Periodontitis may negatively impact facial aesthetics, functionality, and nutrition, affecting an individual’s quality of life and psychological well-being [2]. The management of periodontitis is essential for oral and overall health since there is growing evidence connecting periodontal disease to many systemic conditions, including cardiovascular disease, diabetes, respiratory disease, and adverse pregnancy outcomes [3]. These results underscore the need for efficacious strategies for treatment to mitigate periodontal inflammation and tissue deterioration [4]. The conventional treatment for periodontitis is scaling and root planing (mechanical debridement) to eliminate plaque and calculus in conjunction with antibiotic medication [5]. Nonetheless, the growing issue of antibiotic resistance, along with the need for novel, effective, and sustainable treatment modalities, has compelled researchers to investigate other therapeutic strategies, such as ozone therapy [6,7]. (O_3_) is a triatomic oxygen molecule with strong oxidative characteristics that have a substantial antibacterial impact. O_3_ not only eradicates bacterial cell walls but also diminishes inflammation and facilitates tissue healing, making it a viable option for periodontal therapy [8]. Ozone may cure infections and facilitate healing in periodontal disease by eliminating harmful bacteria and reducing oxidative stress [9]. Recent investigations have validated the use of O_3_ as a therapeutic adjunct in dentistry [10,11]. Evidence indicates that it may impede biofilm development and improve fibroblast activity in vitro; hence, ozone is regarded as an essential instrument in regenerative periodontal treatment [12,13]. Furthermore, periodontal cell lines serve as valuable models in periodontal research for examining cellular responses and molecular processes [14]. These cell lines provide a controlled environment to examine the impacts of pharmaceutical agents and facilitate the comprehension of biological mechanisms, including proliferation, differentiation, and apoptosis [15]. Recent advancements in cell culture techniques, particularly studies on the interaction between O_3_ and periodontal ligament stem cells (PDLSCs), have shown their ability to enhance cellular differentiation and matrix formation, both crucial for tissue regeneration [16]. Consequently, periodontal cell lines have emerged as valuable resources in the research of novel pharmaceuticals, the advancement of pharmacological investigations, and the examination of tissue engineering approaches intended to regenerate lost or damaged periodontal tissues [17]. These cellular models are under investigation and may result in enhancements to diagnostic methodologies and therapeutic strategies. Research demonstrated that O_3_ treatment promotes the proliferation and migration of periodontal ligament fibroblasts under an oxidative stress environment, elucidating its molecular mode of action [18,19]. These results contribute to the accumulating data endorsing the use of O_3_ in addressing inflammatory and bacterial infections, as well as facilitating regenerative healing in periodontitis [20]. This review looks into the impact of O_3_ on periodontal cell lines concerning cell viability, proliferation, and differentiation, as well as its use as an adjunctive therapy in the management of periodontal disease.

### 1.1. Mechanisms of Ozone Action

O_3_ therapy is presented as a new way to treat periodontal diseases because of its strong antimicrobial activity. It can be an alternative or supplementary method to conventional antimicrobial agents [21]. O_3_, a strong oxidizing agent, is effective against all types of microbial pathogens, including bacteria, viruses, fungi, and protozoa, and can be used to decrease the microbial load in periodontal pockets and may lead to better clinical results. Its interaction with the host eukaryotic cells is also involved in immunomodulation and tissue regeneration, which makes it a good candidate for therapy [22]. Recent advancements in research have expanded our understanding O_3_’s mechanisms, particularly in distinguishing its effects on prokaryotic versus eukaryotic cells and exploring microbial resistance mechanisms [23,24,25,26,27]. (Table 1).

### 1.2. The Multifaceted Mechanisms and Clinical Applications of Ozone Therapy in Periodontal Treatment

**Figure 1:**
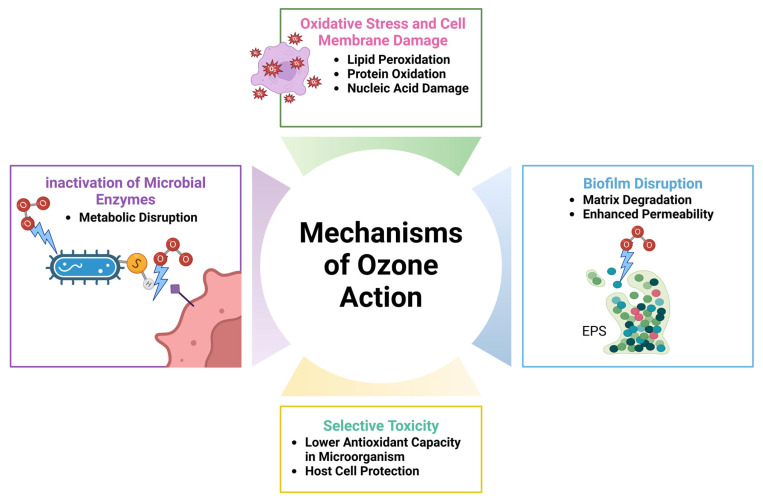
Highlights the mechanisms of O_3_ action in microbial control and tissue repair. Ozone induces oxidative stress, leading to lipid peroxidation, protein oxidation, and nucleic acid damage, compromising cell membranes. It disrupts microbial biofilms by degrading the matrix and enhancing permeability. O_3_ inactivates microbial enzymes, causing metabolic disruption. Its selective toxicity targets microorganisms with lower antioxidant capacity, sparing host cells and enhancing their protection. These combined actions underscore ozone’s therapeutic potential in reducing microbial burden and promoting healing.

Oxidative stress, in which reactive oxygen species (ROSs) such as peroxides, superoxides, and hydroxyl radicals are produced upon contact with microbial cells, is the main antibacterial mechanism of ozone [28]. ROSs provoke lipid peroxidation in microbial membranes, undermining their structural integrity and enhancing permeability. This results in osmotic imbalances and cellular lysis. Furthermore, ROSs induce protein oxidation, inhibiting enzyme functionality essential for microbial metabolism, and inflict damage on nucleic acids via strand breakage and mutations, thereby compromising microbial reproduction and survival [29,30] (Figure 1).

The impacts of O_3_ impact biofilms, which are dense microbial populations that exhibit resistance to standard treatments [31]. O_3_ disrupts the biofilm matrix by degrading extracellular polymeric compounds, increasing biofilm permeability, and facilitating the penetration of antimicrobial drugs to eradicate embedded bacteria [32]. Moreover, O_3_’s selective toxicity mostly affects bacteria with diminished antioxidant defenses while preserving host cells, which are equipped with strong antioxidant systems like superoxide dismutase and catalase [33]. (Figure 1). 

**Figure 2:**
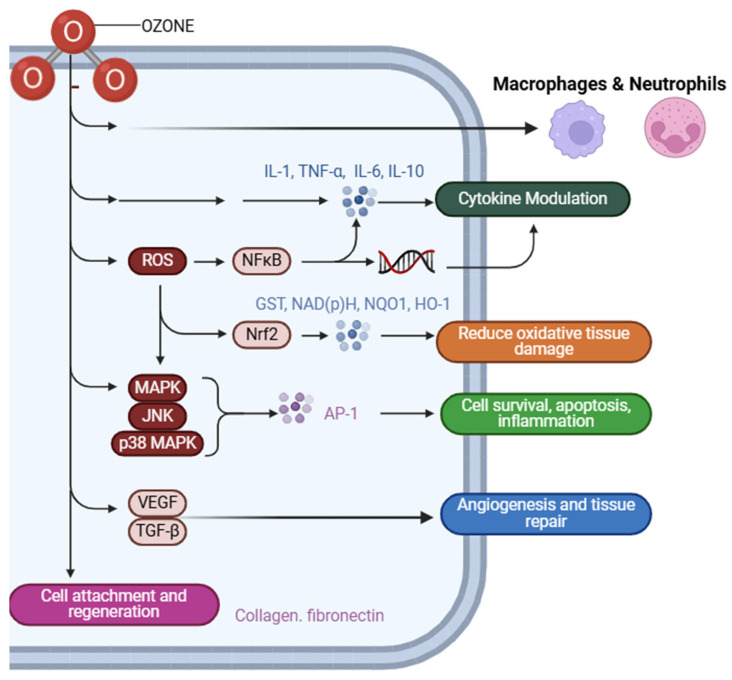
Illustrates the cellular and molecular mechanisms activated by O_3_ therapy. ROS stimulate NF-κB and MAPK pathways, leading to cytokine modulation (IL-1, TNF-α, IL-6, IL-10) and promoting cell survival, apoptosis, and inflammation. Concurrently, the Nrf2 pathway enhances antioxidant responses, reducing oxidative tissue damage through upregulation of GST, NAD(P)H, NQO1, and HO-1. VEGF and TGF-β mediate angiogenesis and tissue repair, while collagen and fibronectin contribute to cell attachment and regeneration. The interplay of macrophages and neutrophils further supports immune modulation and tissue healing.

In eukaryotic cells, ozone has immunomodulatory effects by affecting critical signaling pathways and facilitating tissue healing. O_3_-induced reactive oxygen species activate the Nrf2 pathway, resulting in the transcription of antioxidant and cytoprotective genes, including glutathione S-transferase and heme oxygenase-1, which safeguard host cells from oxidative damage [34]. The MAPK and (Extracellular Signal-Regulated Kinase (ERK), c-Jun N-terminal Kinase (JNK), and p38 Mitogen-Activated Protein Kinase (p38) pathways simultaneously modulate cellular responses to stress, inflammation, and apoptosis, maintaining equilibrium between cell survival and regulated cell death. For instance, JNK and p38 facilitate the removal of injured cells by apoptosis, while ERK promotes cell survival and aids in tissue regeneration [35]. (Figure 2).

O_3_ amplifies the function of macrophages and neutrophils in immune cells, which are essential elements of the innate immune system. It enhances these cells’ phagocytic activity and the synthesis of antimicrobial agents, including reactive nitrogen species and cytokines, therefore augmenting the host’s capacity to battle periodontal infections. O_3_ influences dendritic cells, enhancing antigen presentation and promoting adaptive immune responses [36,37]. Moreover, O_3_ regulates cytokine synthesis, equilibrating pro-inflammatory and anti-inflammatory responses. It downregulates cytokines such as Interleukin-1 Beta (IL-1β), Interleukin-6 (IL-6), and Tumor Necrosis Factor Alpha (TNF-α) to mitigate excessive inflammation while enhancing Interleukin-10 (IL-10), an anti-inflammatory cytokine, to foster an environment favorable to healing and tissue repair [26,38,39]. (Figure 2). 

O_3_ furthermore influences gene expression associated with inflammation, apoptosis, and repair. It first enhances the production of pro-inflammatory cytokines to facilitate pathogen elimination [40]. Nonetheless, its stimulation of the Nrf2 pathway mitigates this reaction, resulting in the transcription of anti-inflammatory genes and diminishing collateral tissue damage. Moreover, O_3_ activates growth factors such as Vascular Endothelial Growth Factor (VEGF) and Transforming Growth Factor Beta (TGF-β), facilitating angiogenesis, collagen synthesis, and extracellular matrix creation, which are critical for wound healing and the regeneration of periodontal tissues [41]. Through the activation of essential pathways of signaling, such as the Nrf2, ozone regulates the transcription of genes involved in oxidative stress response, inflammation, and tissue repair. The Nrf2 pathway promotes the expression of antioxidant enzymes like glutathione S-transferase (GST) and heme oxygenase-1 (HO-1), which mitigate oxidative damage and enhance cellular resilience [42,43].

The MAPK pathways further mediate cellular stress responses, with the ERK pathway promoting cell survival and tissue regeneration, while the JNK and p38 pathways facilitate controlled apoptosis to eliminate damaged or infected cells [44]. Controlled cell death, or apoptosis, is a crucial aspect of ozone’s effect on gene expression. O_3_ regulates apoptosis by activating MAPK pathways, including JNK and p38 MAPK, which upregulate pro-apoptotic genes such as *Bax* and *caspases.* This process ensures the removal of damaged or infected cells, preventing their persistence and promoting tissue homeostasis. At the same time, the ERK pathway is activated, enhancing the expression of anti-apoptotic genes like *Bcl-2* [45]. This action prevents premature apoptosis of healthy cells, preserving their functionality and contributing to tissue stability. By carefully modulating apoptotic processes, O_3_ ensures that only compromised cells are eliminated while supporting the survival of essential ones, facilitating repair and regeneration [46].

O_3_’s ability to promote tissue repair is mediated through the upregulation of growth factors and extracellular matrix proteins. For instance, O_3_ stimulates the expression of VEGF and TGF-β, which are vital for angiogenesis, fibroblast proliferation, and collagen. synthesis. These processes enhance wound healing and facilitate the regeneration of periodontal tissues [47]. Additionally, O_3_ upregulates genes responsible for the production of matrix proteins such as collagen and fibronectin, which provide the structural framework necessary for tissue remodeling and regeneration [47]. These processes facilitate the restoration of periodontal architecture and functionality, emphasizing O_3_ ’s role in periodontal tissue recovery [48]. The literature has offered profound insights into O_3_ ’s impact on gene expression. Studies indicate that ozone-induced Nrf2 activation boosts antioxidant defenses and promotes the expression of genes related to extracellular matrix synthesis, enhancing cellular resilience and facilitating tissue healing [49,50].

O_3_’s antibacterial, immunomodulatory, and regenerative capabilities make it a notable adjunct in periodontal therapy. O_3_ therapy has dual advantages by improving immune cell functionality, regulating the inflammatory response, and facilitating tissue regeneration, thus aiding in infection control and healing enhancement [9,51].

Nevertheless, individualized application tactics are essential to combat the increasing rates of microbial resistance and to attain improved clinical results, hence enhancing the treatment of periodontal diseases [52]. Recent studies have further elucidated the immunomodulatory effects of O_3_. These investigations corroborate its capacity to activate the immune system without inflicting considerable harm to surrounding tissues, providing novel insights into its use in clinical environments [38,53]. According to Ma et al., periodontal tissues offer a protective–regenerative function via ozone-induced Nrf2 activation, which decreases oxidative stress and stimulates cellular proliferation [54].

### 1.3. Periodontal Cell Line in Periodontal Disease

Periodontal cell lines are laboratory-cultured cells derived from the tissues that comprise the periodontium, including the gingiva, periodontal ligament, cementum, and alveolar bone [55]. These cell lines offer an essential framework for examining the pathogenesis of periodontal disease and developing new therapeutic strategies [55].

**Figure 3:**
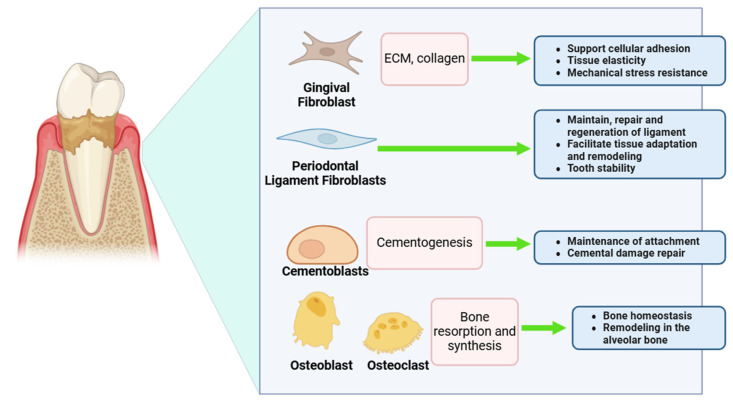
Illustrates the roles of different cells in periodontal tissue health and repair. Gingival fibroblasts produce (ECM) and collagen, essential for cellular adhesion, tissue elasticity, and mechanical stress resistance. Periodontal ligament fibroblasts maintain, repair, and regenerate ligament tissue, promoting adaptation and tooth stability. Cementoblasts support cementogenesis, ensuring attachment maintenance and cemental damage repair. Osteoblasts and osteoclasts coordinate bone resorption and synthesis, maintaining bone homeostasis and remodeling in the alveolar bone. Together, these cellular interactions preserve periodontal structure and function.

Each cell type within the periodontium plays a distinct role in maintaining the integrity of periodontal tissues [55]. Gingival fibroblasts are essential for producing extracellular matrix components and collagen, which contribute to the resilience and repair of gingival tissues while defending against bacterial invasion and inflammation. Periodontal ligament fibroblasts anchor teeth to the surrounding alveolar bone, mediate responses to mechanical forces, and facilitate tissue repair and regeneration [56]. Cementoblasts are responsible for forming cementum, the mineralized tissue that anchors the periodontal ligament to the tooth root, while osteoblasts and osteoclasts regulate alveolar bone remodeling, ensuring structural stability and adaptation to physiological or pathological changes (Figure 3) [57,58]. Recent studies have utilized periodontal cell lines, including gingival fibroblasts, periodontal ligament (PDL) cells, and stem cells derived from periodontal tissues, to explore the molecular mechanisms driving these pathological processes and to create targeted therapies that reflect our evolving understanding of the disease [17,59].

Gingival fibroblasts have a dual role in the inflammatory phase of periodontal disease. They not only participate in the inflammatory process but also produce pro-inflammatory cytokines like IL-6 and TNF-α in response to microbial pathogens. Additionally, these fibroblasts are vital for the integrity and repair of tissue through the production of extracellular matrix (ECM) components, including collagen and fibronectin, which are essential for maintaining tissue structure [60]. Understanding how these cells alternate between destructive and reparative functions has revealed new therapeutic targets for regulating their behavior throughout disease progression [60].

PDL cells, which anchor teeth to the alveolar bone, are a key focus of periodontal research. These cells are directly affected during disease progression, as inflammatory mediators diminish their ability to maintain periodontal attachment and support homeostasis [61]. Adaptation and repair under favorable settings have been shown in recent research employing PDL cell lines, which have focused on their involvement in reacting to mechanical stress and inflammatory stimuli [61,62]. New possibilities for periodontal regeneration have emerged as a result of cellular and molecular biology discoveries that show PDL cells may develop into osteoblasts and cementoblasts. Molecular mechanisms such as Wnt, MAPK, and TGF-β signaling control this differentiation and are now being investigated as potential therapeutic targets for repairing periodontal tissues that have been injured [61,63].

A fundamental aspect of regenerative periodontal therapy is stem cells derived from periodontal tissues, known as PDLSCs. Osteoblasts, cementoblasts, and fibroblasts are three critical cell types involved in periodontal tissue regeneration, and these multipotent cells have shown the ability to differentiate into all three [64]. The potential of PDLSCs to upregulate mineralization-related genes, such as alkaline phosphatase and osteocalcin, has been linked to enhanced bone and ligament repair in preclinical models [65]. Alveolar bone loss and periodontal attachment issues may be effectively addressed through tissue engineering techniques made possible by the regenerative capabilities of these cells. These techniques encompass scaffold-based treatments and the administration of bioactive molecules [66]. By incorporating these discoveries into our understanding of the molecular and cellular processes that contribute to periodontal disease, periodontal cell lines are a priceless tool for bridging the gap between the laboratory and the clinical application [65,66]. These cell models have not only advanced our understanding of disease pathogenesis but have also expedited the development of innovative treatments aimed at regulating inflammation, restoring tissue integrity, and preventing disease recurrence [67]. With ongoing advancements in cellular and molecular research, periodontal cell lines continue to lead efforts to combat periodontal disease and enhance patient outcomes [67] (Table 2).

### 1.4. Effect of Ozone on Periodontal Cell Lines

O_3_ therapy, recognized for its antimicrobial, anti-inflammatory, and oxidative stress-modulating properties, has demonstrated promising results in enhancing the functional capabilities of PDLSCs [68]. For example, O_3_ exposure has been linked to increased proliferation and differentiation of PDLSCs into osteogenic and fibroblastic lineages, facilitating periodontal regeneration [69]. Moreover, ozone has been shown to upregulate the production of growth factors and extracellular matrix components, improving the regenerative microenvironment necessary for tissue repair [70]. These findings indicate that O_3_ therapy could serve as a valuable adjunct in periodontal treatments aimed at harnessing the regenerative capacity of PDLSCs [69,70]. To maximize the therapeutic outcomes of O_3_ while minimizing potential adverse effects, careful attention must be given to the dosage and application duration. Based on the literature, an O_3_ concentration of 10–40 µg/mL applied for short intervals (30–60 s per treatment cycle) has been shown to stimulate cellular proliferation and differentiation without inducing cytotoxicity [71]. Prolonged or excessive ozone exposure, however, can lead to oxidative damage, highlighting the need for precise control in clinical applications [72].

Implementing these advancements to restore damaged tissues and regenerate periodontal structures offers hope for improved outcomes in patients with periodontitis.

### 1.5. Therapeutic Applications of Ozone in Periodontal Therapy

The ability of O_3_ to selectively target pathogenic microorganisms, modulate host immune responses and promote tissue repair makes it a promising tool for managing periodontal disease, characterized by chronic inflammation and microbial biofilms [73]. Numerous studies have highlighted O_3_’s effectiveness in periodontal therapy, particularly for its role in reducing bacterial loads in periodontal pockets. Controlled O_3_ application has demonstrated significant reductions in periodontal pathogens such as *Porphyromonas gingivalis* and *Tannerella forsythia*, which are key contributors to disease progression [74,75,76,77,78]. This targeted antimicrobial action not only disrupts biofilms but also prevents recolonization, making it an effective adjunct to scaling and root planing (SRP), the gold standard in periodontal therapy [77].

In addition to its antimicrobial effects, ozone has been shown to modulate inflammatory responses in periodontal tissues. It reduces pro-inflammatory cytokines IL-1β, IL-6, and TNF-α while enhancing anti-inflammatory mediators such as IL-10 [19,38]. This dual action contributes to a balanced immune response, which is critical for mitigating tissue destruction and promoting healing in periodontal therapy [79].

While O_3_ therapy offers several advantages, it is not without limitations. The lack of long-term clinical trials evaluating its efficacy in comparison to standard treatments like chlorhexidine and SRP limits its widespread adoption [80]. Additionally, adverse effects such as transient tissue irritation have been reported, emphasizing the importance of adhering to established protocols. Nevertheless, O_3_’s unique ability to combine antimicrobial, anti-inflammatory, and regenerative properties positions it as a valuable tool in the comprehensive management of periodontal disease [81].

Integrating O_3_ therapy into periodontal practice provides a promising opportunity to enhance therapeutic outcomes, particularly in cases of refractory periodontitis or patients with contraindications to conventional antimicrobials [80,81]. Future research should focus on standardized protocols, comparative studies with established treatments, and patient-reported outcomes to solidify O_3_’s role in evidence-based periodontal care.

### 1.6. Therapeutic Applications of Ozone in Dentistry

#### 1.6.1. Endodontic Treatment

While this review is primarily about periodontal therapy, it is important to note ozone’s use in endodontics as well. The problem of eliminating biofilms in a complex root canal structure has made ozone a potential additional tool in root canal disinfection [82]. Ozone can penetrate biofilm matrices and has very strong antimicrobial action that is very efficient in reducing bacterial numbers, including resistant species like Enterococcus faecalis [83,84,85]. It has been shown that including ozone in endodontic treatments lowers the chance of reinfection, enhances the success rate of treatments, and benefits patients. For instance, Sinha, et al. found that microbial numbers were greatly reduced after ozone treatment than with conventional chemical irrigants [86].

#### 1.6.2. Oral Surgery

Ozone therapy in oral surgery is useful in preventing postoperative infections, enhancing wound healing, and increasing patient comfort. The antimicrobial and regenerative properties of ozone are particularly valuable in surgical procedures that involve tissue resection or implant placement [87,88].

Clinical studies have shown that ozone decreases the occurrence of postoperative problems by decreasing bacterial numbers and enhancing the rate of tissue regeneration. For example, the application of ozonated oils has been found to stimulate angiogenesis and improve fibroblast activity, thus enhancing the healing rate and reducing the healing time. Furthermore, the use of gaseous ozone in implant surgeries has been linked with better osseointegration, providing additional advantages in surgical outcomes [89,90,91,92].

#### 1.6.3. Balancing Ozone Exposure in Therapeutic Applications

Ozone therapy presents a powerful tool in periodontal treatment, offering significant antimicrobial, anti-inflammatory, and regenerative benefits. However, its therapeutic efficacy depends heavily on precise dosage control, localized application, and a clear understanding of its cellular mechanisms. This balance ensures maximum therapeutic outcomes while minimizing potential cytotoxic effects [10].

#### 1.6.4. Controlled Dosage

Ozone’s therapeutic efficacy and safety hinge on carefully controlling dosage and exposure time [78].

According to research, maintaining ozone concentrations within the therapeutic window is essential for optimizing its benefits without overtaxing the tissue’s antioxidant mechanisms. For example, studies have shown that gaseous ozone concentrations of 10–40 µg/mL and exposure durations of 30–60 s effectively generate antibacterial activity while preserving cellular viability. Ozone may overcome cellular defenses and have cytotoxic effects at higher concentrations due to its ability to induce excessive oxidative stress. On the other hand, its hormetic benefits, which boost cellular resilience and repair processes, are enhanced at regulated, lower dosages [71,93].

In periodontal treatment, for example, clinicians must follow precise, well-established protocols to maximize results and minimize risks. Implementing real-time concentration monitoring and other innovations in ozone delivery systems allows practitioners to apply ozone consistently and safely [94].

#### 1.6.5. Localized Application

Localized application of ozone is a critical strategy to enhance its specificity and reduce systemic exposure. In periodontal therapy, targeted ozone delivery into periodontal pockets ensures concentrated antimicrobial action at the site of infection without affecting surrounding healthy tissues [95]. Recent clinical studies demonstrate that localized ozone application reduces bacterial counts within periodontal pockets while improving clinical attachment levels and pocket-depth reduction [20,96].

Beyond periodontal therapy, localized ozone application has shown efficacy in various clinical conditions, including peri-implantitis and endodontic infections [97,98]. For example, ozonated water irrigation has been used successfully in endodontic therapy to reduce biofilm-associated pathogens, while topical ozone application has demonstrated wound-healing benefits in oral surgical sites [99]. Such studies emphasize the versatility of localized ozone therapy in diverse clinical scenarios, highlighting its ability to target specific areas with precision and minimal adverse effects [96,97,98].

#### 1.6.6. Induction of Protective Cellular Responses

Inducing defensive cellular responses via hormesis is one of the most fascinating elements of ozone treatment. Low-dose ozone exposure triggers adaptive cellular pathways that produce moderate oxidative stress to improve tissue resilience and recovery [93,100]. Crucial to this process is the Nrf2 (Nuclear Factor Erythroid 2-Related Factor 2) pathway, which increases the expression of genes that code for antioxidant enzymes including SOD, catalase, and glutathione peroxidase. Protecting cellular components and aiding in tissue repair, these enzymes fight oxidative stress [101]. 

New studies have shown that different kinds of tissues react differently to oxidative stress, and this has led researchers to focus on the tissue-specific processes of ozone-induced hormesis [102]. For instance, periodontal ligament fibroblasts may increase matrix production and proliferation when exposed to low levels of ozone, yet epithelial cells can demonstrate higher antioxidant activity and less inflammation. These anticipated outcomes may suggest that the hormetic effects of ozone are strongly dependent on the specific tissue type and local microenvironment [103]. By looking into these processes, we may approach the use of ozone in regenerative periodontal treatments from a novel point of view. Through precise dosing and administration, clinicians may tap into ozone’s capacity to promote defensive responses, hasten wound healing, and heighten periodontal tissue regeneration.

## 2. Challenges and Considerations

Despite its potential, ozone therapy faces several challenges that must be addressed for safe and effective integration into clinical practice:

- Standardization of Dosage: One of the primary challenges with ozone therapy is determining the precise concentration and exposure time required for optimal effects on periodontal tissues. While lower ozone concentrations are associated with beneficial effects on cellular proliferation and antimicrobial activity, higher concentrations can be cytotoxic, causing damage to host tissues. Rigorous research is required to establish dosage parameters that balance therapeutic efficacy with cellular safety, ensuring reproducible and predictable results in clinical settings.
- Development of Effective Delivery Systems: To harness the benefits of ozone therapy, it is essential to design and develop advanced delivery systems that allow for precise, controlled, and targeted application. Effective delivery methods would ensure that ozone reaches specific areas within periodontal pockets or damaged tissues without impacting surrounding healthy structures. Various forms, such as gaseous ozone, ozonated water, and ozone oils, have been explored, yet more research is needed to identify the most efficient and practical application methods that maximize therapeutic impact while minimizing systemic exposure.
- Assessment of Long-term Effects: While short-term benefits of ozone therapy in periodontal treatment have been documented, there is a lack of extensive longitudinal studies that evaluate its long-term impact on periodontal tissues and overall oral health. Understanding the effects of repeated ozone applications on tissue integrity, cellular health, and periodontal stability is crucial to determining its safety and efficacy over extended periods. Long-term studies will provide insight into potential risks, cumulative effects, and the possibility of chronic tissue alterations or adaptive responses to ozone exposure.

Further investigation into these areas will be instrumental in overcoming the current challenges associated with ozone therapy, refining its application, and unlocking its full potential in the management and treatment of periodontal disease. Addressing these considerations could establish ozone therapy as a valuable component of comprehensive periodontal care, expanding therapeutic options and enhancing patient outcomes in the field of dental medicine.

## 3. Conclusions

Ozone therapy has emerged as a promising adjunctive treatment in periodontal care, primarily due to its potent antimicrobial properties, as well as its ability to stimulate cell proliferation, differentiation, and modulate inflammatory responses. By leveraging ozone’s capacity to reduce bacterial load, promote healing, and support tissue regeneration, clinicians may enhance the effectiveness of traditional periodontal treatments, potentially reducing treatment duration and improving clinical outcomes. Current evidence points to significant benefits of ozone therapy in periodontal applications; however, the need for a more comprehensive understanding of its biological mechanisms and optimization of its therapeutic protocols remains essential. Establishing evidence-based guidelines for ozone application could pave the way for its widespread adoption in periodontal therapy, offering an innovative approach to managing periodontal diseases and improving patient outcomes.

## Figures and Tables

**Table 1:** Comparison of ozone’s effects on prokaryotic and eukaryotic cells, highlighting targeted structures, mechanisms, and clinical implications.

Aspect	Prokaryotic Cells	Eukaryotic Cells
Targeted Structures	Cell membrane lipids, proteins, and DNA	Host cell signaling pathways, antioxidant systems, and growth factors
Mechanism of Action	Lipid peroxidation, protein oxidation, nucleic acid damage	Activation of Nrf2 and MAPK pathways; modulation of cytokine and growth factor expression
Antioxidant Defense	Limited antioxidant defenses; higher susceptibility to ROS	Robust antioxidant systems, including enzymes like superoxide dismutase and catalase
Effects on Cell Survival	Disruption of cellular integrity, leading to cell lysis and death	Promotes cell survival via ERK pathway; induces controlled apoptosis via JNK and p38 pathways
Effects on DNA	DNA damage through strand breaks and mutations; inhibits replication	Minimal DNA damage due to enhanced antioxidant response; protects genomic stability
Resistance Mechanisms	Upregulation of oxidative stress defense genes in biofilm-associated pathogens	No significant resistance noted; mechanisms focus on repair and modulation
Clinical Implications	Effective for biofilm disruption and microbial eradication; may require combination therapies to address resistance	Promotes tissue repair and regeneration; enhances host immune modulation

**Table 2:** Highlights the diverse research applications of periodontal cell lines, encompassing pathophysiological mechanisms, therapeutic development, tissue engineering, and genetic studies. Each application provides insights and potential outcomes for advancing periodontal disease management and treatment strategies.

Research Area	Applications	Potential Outcomes
Pathophysiological Mechanisms	Study cellular responses to bacterial infections, inflammation, and oxidative stress.	Identify therapeutic targets to halt or reverse periodontal disease progression.
Therapeutic Development	Test new drugs and therapeutic strategies in vitro before animal models or clinical trials.	Improve drug safety and effectiveness for potential periodontal treatments.
Tissue Engineering and Regeneration	Develop biocompatible scaffolds and biomaterials to support periodontal tissue regeneration.	Enhance tissue recovery and repair periodontal defects with innovative regenerative strategies.
Genetic Studies	Investigate the roles of specific genes in periodontal health and disease.	Highlight genetic risk factors and develop gene therapies for periodontal disease management.
